# Risk factors and prevention strategies for complications following thyroid radiofrequency ablation: A review

**DOI:** 10.1097/MD.0000000000044039

**Published:** 2025-08-22

**Authors:** Junli Qiao, Shuyan Li, Zhanbin Cui, Lili Zhang, Mingqiang Han

**Affiliations:** a Department of Head and Neck Thyroid Surgery, Xingtai People’s Hospital, Xingtai, Hebei, China; b Dermatology, The Affiliated Taian City Central Hospital of Qingdao University, Taian, Shandong, China.

**Keywords:** complications, prevention strategies, risk factors, thyroid radiofrequency ablation

## Abstract

Thyroid radiofrequency ablation is a minimally invasive procedure increasingly employed in the management of thyroid nodules. Despite its advantages, postoperative complications remain a concern. This article aims to compile a comprehensive review of the risk factors associated with these complications, and strategies to mitigate them. We systematically reviewed the literature, using rigorous selection criteria, to identify pertinent studies. Our findings underscore several key risk factors (including age, sex, medical history, etc). Concurrently, we illustrate preventive strategies (such as pain, hematoma, cough, etc) demonstrating their potential role in clinical practice. By highlighting these aspects, this review aids clinicians in executing more effective, safer thyroid radiofrequency ablation procedures, and sets a foundation for future research in this domain.

## 1. Introduction

Thyroid diseases are characterized by a high prevalence in adults, and the detection rate has significantly increased in recent years due to the development of diagnostic imaging technologies.^[1]^ Thyroid nodules (TN), a common condition within thyroid diseases, have shown a surprising incidence of 20% to 76%.^[2]^ Additionally, the rates of hyperthyroidism, compressive symptoms, and malignant tumor detection have continued to rise.^[3]^ In the past, total thyroidectomy or thyroid lobectomy were the mainstay treatments for symptomatic and malignant TN. However, the reliance on surgical techniques has led to a significant occurrence of complications (7–19%), highlighting the importance of considering thyroid radiofrequency ablation (RFA) as an emerging treatment method, which has notably reduced the incidence of postoperative complications.^[4]^ Since its introduction into the medical field in 2006, RFA has made significant progress. Its advantages, including minimally invasive nature, minimal scarring, rapid recovery, and minimal risk of thyroid function decline in appropriate patient populations, make it a feasible and effective option for treating thyroid diseases. Initially used for benign conditions such as thyroid cysts and nodules, RFA’s application has expanded to include the treatment of thyroid malignancies as the technology has matured.^[5]^

Deandrea et al conducted a 5-year longitudinal observation of benign thyroid nodule RFA treatment, concluding that RFA can serve as an alternative to surgical excision techniques.^[6]^ Furthermore, the importance of RFA as a treatment option for recurrent thyroid cancer is gradually increasing.^[7]^ However, as a progressive treatment technique, it is crucial to pay attention to the different postoperative complications that may arise from this novel technology.^[8]^ Complications such as vocal cord paralysis, refractory hypocalcemia, bleeding, and infections have been increasingly reported in recent years.^[9–11]^ Controlling post-RFA complications will have a positive impact on patients’ quality of life and treatment outcomes.^[12]^

**Figure 1.**
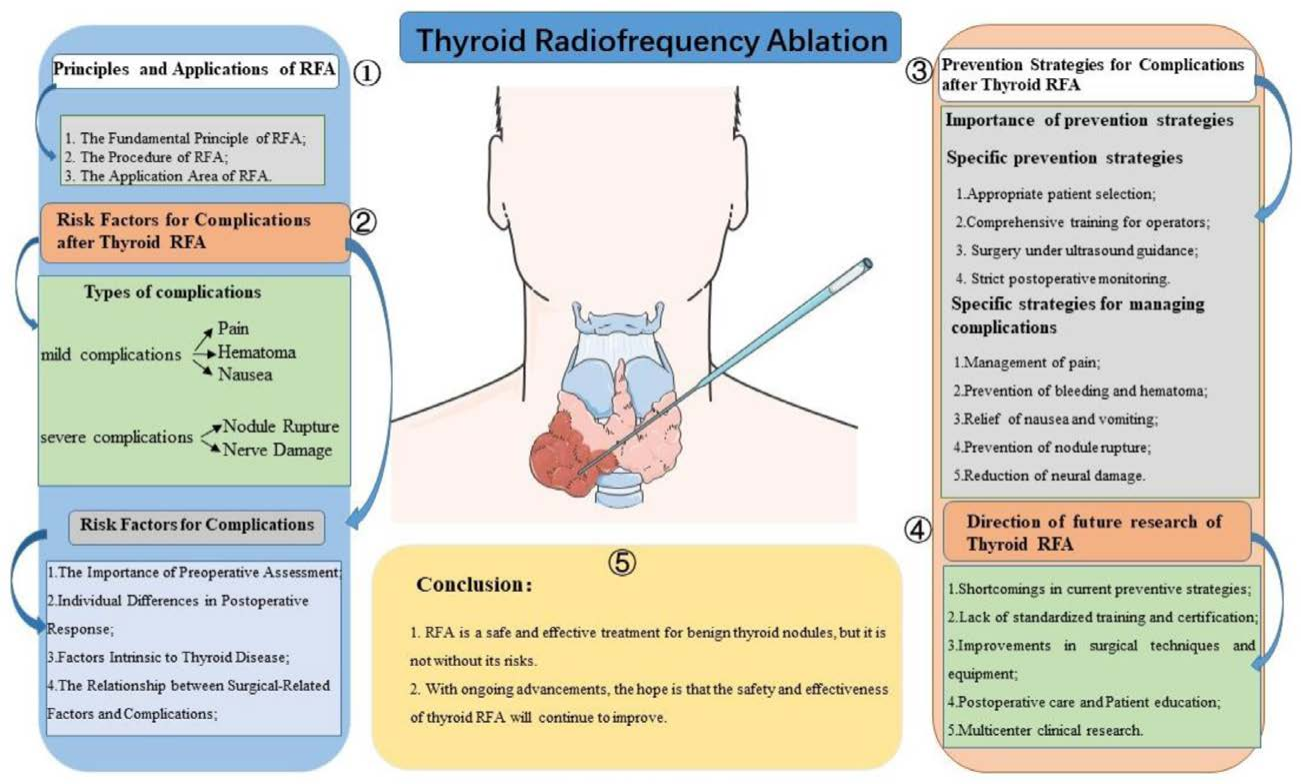
Complications, risk factors, and prevention strategies after thyroid RFA surgery. RFA = radiofrequency ablation.

In this review, we summarize the common post-RFA complications, surgical risk factors, and strategies for managing these complications, aiming to propose future research directions for this technology. We hope that this review will contribute to a deeper understanding of the mechanisms underlying postoperative complications, with the ultimate goal of enhancing the safety and efficacy of thyroid RFA and exploring effective preventive and management strategies to reduce patients’ suffering and adverse effects, thereby advancing the development and application of thyroid RFA further (Fig. 1).

## 2. Principles and applications of RFA

The fundamental principle of RFA involves the conversion of radiofrequency energy into thermal energy through friction between the molecules of the surrounding tissues, leading to coagulative necrosis of the target tissue while preserving the surrounding healthy tissue.^[13]^ RFA is a noninvasive therapeutic technique guided by ultrasound to target TN.^[14]^ Under local anesthesia, a physician uses a fine needle (17–22 gauge) to puncture the nodule, and the tip of the needle emits radiofrequency current.^[15]^ The current generates heat within the nodule, leading to an increase in tissue temperature. When the temperature reaches 60 to 100 °C, evaporating water causes coagulative necrosis, achieving ablation. Throughout the procedure, the physician monitors the temperature and may use a cooling system to prevent overheating.^[16]^ After completion of the ablation, the needle is withdrawn, and the puncture site heals on its own. Following treatment, patients require regular follow-up examinations to assess efficacy and monitor potential complications. This therapy is suitable for treating benign TN that may be symptomatic or carry a malignant risk.^[17]^

RFA is an interventional medical technology primarily used to treat benign or malignant diseases of the heart, lungs, liver, kidneys, thyroid, bone, and soft tissues without the need for surgical excision.^[18]^ It has demonstrated extensive applications in the treatment of benign TN and recurrent thyroid cancer, effectively reducing nodule size, improving symptoms, enhancing cosmetic outcomes, and even achieving tumor necrosis.^[19]^ RFA is used in the treatment of cardiac arrhythmias, such as atrial fibrillation, by heating cardiac tissue with a percutaneously inserted radiofrequency probe to eliminate abnormal circuits.^[20]^ In the liver, RFA is commonly employed to treat small hepatocellular carcinoma by ablating cancer cells through the abdominal wall.^[21]^ In kidney diseases, such as adrenal tumors (e.g., pheochromocytoma), RFA is also utilized for ablation.^[22]^ Furthermore, RFA may also be employed to address benign lung tumors, pulmonary vein stenosis, TN (malignant or at high surgical risk), bone tumors, osteoarthritis, soft tissue tumors, and certain neuropathic pain conditions, such as pain induced by intervertebral disc protrusion.^[23–27]^ During the procedure, physicians select appropriate ablation parameters based on the patient’s specific condition and lesion location to ensure safety and efficacy.^[28]^ This approach represents a minimally invasive and rapidly restorative therapeutic modality but requires the expertise of a specialized medical team for its execution.

## 3. Risk factors for complications after RFA

### 3.1. Types of complications

Surgical complications refer to adverse events and diseases that occur during or after surgery.^[29]^ Accurate definition and classification of surgical complications have a positive impact on postoperative care, complication prevention, and treatment (Table 1).^[33]^ It is important to note that certain events, such as transient voice changes due to lidocaine injection, small hematomas, bleeding, parenchymal edema without symptoms, and tolerable mild pain and discomfort that do not require medication, are considered neither complications nor side effects.^[34]^ Several systematic reviews indicate substantial progress in the safety profile of RFA.^[35]^ For instance, a meta-analysis by Chung et al revealed a postoperative complication rate of 2% to 3%, with a risk of permanent complications or severe injury even lower, at <1%.^[30]^ However, another large systematic review reported higher complication rates, with a rate of significant complications reaching 3.8%, and an overall range of complication occurrence of 0.0% to 16.7%.^[34]^ Although thyroid RFA is generally considered safe, there are still risks of complications, which can be categorized as mild or severe. Mild complications include pain, hematoma, cough, skin burns, and voice changes.^[36]^ Although rare, severe complications may include nodule rupture, severe burns, and vocal cord paralysis.^[37]^

**Table 1:** Structured classification of complications.

Complication type	Incidence	Pathophysiology	Clinical manifestation	Management
Mild complications				
Pain	0.8–15.7%	Thermal stimulation of cervical nerves	Localized discomfort (≤48 hr)	Preoperative lidocaine infiltration, post-op NSAIDs
Hematoma	1–5%	Vessel injury (puncture/thermal)	Neck swelling, dysphagia	Ultrasound-guided drainage (if > 50 mL)
Severe complications				
Nerve injury	0.1–0.5% (permanent)	Thermal/mechanical trauma	Hoarseness (recurrent laryngeal nerve), Horner syndrome	Immediate laryngoscopy, serial neuroimaging
Nodule rupture	<0.3%	Capsular overdistension	Sudden pain, neck mass	Emergency hemostasis (suture/coagulation)

Table format enhances readability; data from.^[30–32]^

#### 3.1.1. Pain

The overall incidence of pain ranges from 0.8% to 15.7%.^[31]^ However, the occurrence of pain is an inevitable aspect of all surgical procedures. The smaller incisions made through skin cuts or puncture needles in RFA, compared to non-minimally invasive techniques, evidently mediate a milder and shorter pain process.^[38]^ Consequently, the number of patients experiencing pain that can be defined as a complication is not as high as anticipated.^[39]^

#### 3.1.2. Hematoma

Hematomas following thyroid RFA are typically caused by vascular rupture due to cutting or thermal damage to thyroid tissue during the surgical procedure, and may occur in the surgical area or spread to other areas of the neck.^[40]^ Symptoms of hematomas may include neck swelling, pain, difficulty breathing, and swallowing, and may require urgent management.^[41]^ The incidence is relatively low but not rare, occurring in approximately 1% to 5% of patients, depending on nodule size, location, and surgical technique.^[42]^ Risk factors include older age, coagulation disorders, longer surgical duration, and nodules located close to major blood vessels.^[14]^ Diagnosis is usually achieved through ultrasound examination, with hematomas possibly presenting as areas of uneven echoes, sometimes requiring further imaging studies. Treatment may involve natural absorption for mild hematomas, but for larger hematomas or severe symptoms, drainage or surgical removal may be necessary, and anticoagulant therapy could be used for prevention or treatment.^[43]^ Preoperative assessment, appropriate surgical technique selection, surgical duration control, and postoperative care and observation all help to reduce the risk of hematomas.^[44]^

#### 3.1.3. Nausea

Postoperative nausea is common and can stem from various factors, including anesthesia drugs within several hours post-surgery, which is a normal physiological reaction that gradually diminishes.^[45]^ Surgical trauma and the body’s stress response to trauma may also cause nausea, which may be exacerbated by side effects of painkillers and antibiotics.^[46]^ Anxiety, dehydration (due to reduced water intake following nausea), and hypoglycemia (due to inadequate postoperative eating) can also induce nausea.^[47–49]^ Individual differences can influence the response to surgery, with certain individuals being more sensitive to specific factors.^[50]^ Approaches to managing post-thyroid RFA nausea include ensuring hydration, providing small and frequent meals, avoiding an empty stomach, prescribing antiemetic drugs for severe nausea, creating a quiet and nonirritating environment, and offering psychological support.^[51–53]^ Overall, postoperative nausea is usually temporary, but if it persists severely or is accompanied by other symptoms, prompt medical attention is necessary.

#### 3.1.4. Nodule rupture

Rupture of TN during RFA surgery is due to thermal energy causing structural damage and wall rupture of the nodules, possibly accompanied by bleeding.^[54]^ Risk factors include nodules located near the capsule, large volume, high hardness, and technical issues during surgery.^[55]^ Symptoms may include sudden neck pain, swelling in the thyroid area, pain radiating to the ear, throat, or shoulder, possibly with bloody secretions, and swallowing difficulty, which may affect breathing in severe cases.^[56,57]^ Diagnosis typically involves ultrasound, which may show uneven echoes or hypoechoic areas, possibly requiring further imaging. Once diagnosed, close monitoring for signs of bleeding is necessary, with significant bleeding possibly requiring emergency intervention such as puncture or surgical hemostasis.^[58]^ If infection occurs, antibiotic treatment is essential. Prevention measures include preoperative assessment, appropriate technique selection, surgical duration control, and precise operation. Postoperative care and observation are also crucial. Regular follow-up may be necessary after nodule rupture to monitor the risk of bleeding and infection and to assess the need for further treatment.^[59]^ Although rupture of TN after RFA is rare, timely identification and management are crucial to prevent complications from worsening.^[54]^ Close monitoring by a specialized postoperative team is essential to ensure safety.

#### 3.1.5. Nerve damage

Nerve damage caused by RFA mainly includes recurrent laryngeal nerve paralysis, brachial plexus nerve paralysis, spinal accessory nerve damage, and Horner syndrome.^[60–62]^ Recurrent laryngeal nerve paralysis is one of the main complications faced by patients undergoing RFA treatment, typically leading to hoarseness, although in most cases, this symptom is temporary and resolves within 2 to 3 months.^[32]^ A very small number of patients may experience permanent voice changes.^[63]^ Brachial plexus nerve damage has been reported in a multicenter study.^[64]^ Due to its deeper anatomical location, the likelihood of brachial plexus nerve injury is not high, resulting in an extremely low incidence of this complication. However, reported real-life cases suggest that surgeons should be vigilant about this high-risk complication, which can cause a series of impairments such as sensory abnormalities, muscle weakness or paralysis, muscle atrophy, and loss of sensation. Another reported complication arising from deep cervical nerve damage is sympathetic nerve damage. Similar to brachial plexus nerve damage, sequelae from sympathetic nerve damage (i.e., Horner syndrome) are also rare, with patients experiencing typical ptosis, anhidrosis, and miosis persisting for more than 6 months without recovery.^[65]^ Three cases of spinal accessory nerve damage were reported by Kim et al.^[66]^ This nerve extends from the brainstem, emerges from the base of the skull, then runs down the neck and connects to the motor nerve roots of the spinal cord. Unlike the aforementioned nerve injuries, the spinal accessory nerve is located more superficially and requires additional attention during surgery.^[67]^

### 3.2. Risk factors for complications

Risk factors for complications encompass inadequate operator experience, size and location of TN, overall health status of the patient, and a history of prior thyroid surgeries.^[68]^ Large nodules, those located near the recurrent laryngeal nerve or trachea, and patients with a tendency for bleeding are at an increased risk of complications.^[69]^

#### 3.2.1. The importance of preoperative assessment

The assessment of risk factors is a crucial step before RFA and plays a pivotal role in the entire process. By comprehensively considering the patient’s baseline characteristics, medical history, clinical manifestations, and other diagnostic results, identifiable risk factors can be recognized.^[70]^ Preoperative assessment aids clinicians in gaining a better understanding of the patient’s overall health status, thereby enabling more accurate assessment of the risk of postoperative complications. Accurately assessing a patient’s risk factors helps in selecting suitable treatment plans and timely implementation of preventive measures to reduce the incidence of complications.^[71]^

#### 3.2.2. Individual differences in postoperative response

Different patient characteristics have varying impacts on the risk of post-thyroid RFA complications. Factors such as age, gender, and medical history can influence the patient’s postoperative recovery and the incidence of complications.^[72]^ For instance, age is a significant influencing factor, with some studies indicating a higher risk of postoperative complications with increasing age.^[73]^ Gender may also have a certain impact on the risk of complications, with research revealing that female patients are more prone to specific complications compared to male patients.^[74]^ Corresponding studies have been conducted to develop risk assessment strategies for specific patient groups. For example, a study focusing on age-related factors found a significantly higher incidence of postoperative complications in patients aged 60 and above, underscoring the need for heightened attention to risk assessment and intervention within this specific age group.^[75]^ In addition to patient-related factors, surgical procedures and equipment selection also significantly influence the risk of post-thyroid RFA complications. Standardized surgical techniques and key operational points are paramount in reducing the occurrence of complications.^[76]^ For example, research has highlighted that complete ablation of TN and avoidance of inadvertent damage to adjacent tissues can significantly reduce the risk of complications.^[77]^ Moreover, device selection is also a critical factor. Different devices possess distinct operational characteristics and risks, hence selecting appropriate equipment can lower the incidence of postoperative complications. Studies have demonstrated that using RFA devices with higher safety and controllability can reduce the risk of postoperative complications.^[17]^

The regulation of the risk of postoperative complications by patient individual differences is an important area of research.^[78]^ Individual factors such as the immune system and metabolic status are believed to be closely related to the risk of postoperative complications.^[79]^ By exploring the regulatory role of these individual differences in complications, researchers can better understand the characteristics of patient groups and provide a basis for the prevention and intervention of postoperative complications.^[80]^ In this regard, the importance of individualized interventions has also been emphasized formulating targeted treatment plans based on the individual characteristics of patients.

#### 3.2.3. Factors intrinsic to thyroid disease

The risk factors of different thyroid lesion characteristics (such as size, location, structure, etc) have an impact on complications. A multitude of studies have reviewed these factors, analyzing their mechanisms and clinical significance in postoperative complications. For example, some studies have found a correlation between the size of TN and their malignancy degree, as well as the incidence of postoperative hypocalcemia.^[81]^ Additionally, the location of thyroid lesions in the anatomical structures of the neck has been proven to be associated with the risk of postoperative complications.^[82]^ Therefore, understanding the relevant risk factors of different thyroid lesion characteristics is crucial for predicting the risk of complications and formulating individualized treatment plans. The impact of patient individual differences on complications.

#### 3.2.4. The relationship between surgical-related factors and complications

In addition to individual factors, surgical-related factors also significantly influence the risk of postoperative complications. Surgical techniques, operative procedures, and postoperative care may all be potential factors contributing to the occurrence of complications.^[83]^ Therefore, researchers have analyzed the impact of these factors during the surgical process on complications. Some studies have proposed strategies to improve surgical procedures and management to reduce the risk of postoperative complications. For instance, optimizing surgical techniques, adhering to strict operation standards, and improving postoperative care are considered to reduce the incidence of complications.^[84,85]^ Moreover, factors such as the experience and proficiency of clinical physicians may also have an impact on surgical outcomes, warranting further research.^[86]^

### 3.3. Prevention strategies for complications after thyroid RFA

#### 3.3.1. Importance of prevention strategies

Given the potential complications, the development and implementation of preventive strategies are crucial. These strategies not only improve patient outcomes and satisfaction but also reduce the overall cost of managing complications.^[87]^

It is imperative to implement strategies to prevent post-RFA complications, including rigorous preoperative assessment, precise surgical procedures, vigilant postoperative monitoring, standardized postoperative care, and provision of necessary postoperative rehabilitation guidance.^[88]^ Prior to surgery, surgeons should conduct comprehensive physical examinations and relevant tests to assess the patient’s surgical suitability and risk, aiming to reduce unforeseen circumstances and the occurrence of complications during surgery. During the surgical procedure, surgeons should minimize damage to surrounding structures as much as possible, avoiding inadvertent injury to vital vessels, the recurrent laryngeal nerve, etc, to ensure the safety and efficacy of the operation. Postoperative monitoring is crucial, and physicians should closely observe changes in the patient’s condition, especially potential complications such as bleeding, hoarseness, etc, and promptly take necessary measures.^[89]^ Standardized postoperative care is also a crucial aspect of preventing complications. Patients need to adhere to the guidance of healthcare professionals, rest, avoid strenuous exercise, and undergo regular follow-up examinations. Additionally, providing necessary postoperative rehabilitation guidance is essential for the patient’s recovery. Physicians should offer relevant rehabilitation guidance, including dietary recommendations and medication management to expedite patient recovery and reduce the risk of complications. In summary, preventing post-thyroid RFA complications requires comprehensive consideration from preoperative assessment to postoperative monitoring and care. Physicians need to communicate fully with patients, informing them of the risks and potential complications of the surgery, and continually update their professional knowledge to enhance the safety and efficacy of the procedure.^[90]^ This comprehensive approach is essential for minimizing the occurrence of post-thyroid RFA complications, ensuring the health and quality of life of patients.

#### 3.3.2. Specific prevention strategies

Preventive strategies encompass appropriate patient selection, comprehensive operator training, the use of ultrasound guidance during surgery, and diligent postoperative monitoring. For high-risk patients, additional measures such as the use of anticoagulants or cooling systems to prevent skin burns may be considered.

#### 3.3.3. Appropriate strategies for managing complications

##### 3.3.3.1. Management of pain

The degree of pain is associated with the thermal power of the puncture needle, the puncture area, the operator’s proficiency, and the number of punctures.^[28]^ Generally, higher needle thermal power leads to more pronounced pain.^[91]^ Preoperative planning tailored to the patient’s specific circumstances, including needle insertion sites, needle thermal power, and puncture areas, is essential. Moreover, it is imperative to require operators to possess proficient technical skills, a clear understanding of the anatomical structure of the puncture site, and to avoid surrounding vessels, muscles, and nerves as much as possible. For smaller nodules, a single-point ablation to achieve complete coverage should be attempted to reduce the number of punctures.^[92]^ When dealing with larger nodules, following the principle of deep to shallow and distant to close is necessary to minimize bleeding and alleviate pain.^[93]^ Pre/postoperative administration of appropriate medications for pain prevention and management, timely wound care, maintaining wound dryness, reducing activity, and avoiding irritants are important, coupled with regular pain assessments and control.

##### 3.3.3.2. Prevention of bleeding and hematoma

Preoperative comprehensive assessment of lesion size, location, and its relationship with surrounding structures using ultrasound and other auxiliary examinations is crucial. This enables thorough planning of the puncture path, while avoiding vessels as much as possible, followed by lesion ablation. Patient cooperation during the ablation process is crucial, and patients are advised to minimize swallowing to avoid inadvertent injury to surrounding blood vessels during the procedure. Precision during the operation is paramount, with attention to reducing the pressure and movement magnitude during needle insertion, and maintaining the stability of the radiofrequency current.^[94]^ Patients taking anticoagulants should discontinue the medication 2 weeks prior to RFA. Early detection and timely management of bleeding and hematoma during postoperative monitoring and care can mitigate the severity of complications.^[95]^

##### 3.3.3.3. Relief of nausea and vomiting

Nausea and vomiting are common post-thyroid RFA complications, attributed to preoperative anxiety, anesthesia method during surgery, and postoperative pain. The use of local anesthesia can reduce the risk of nausea and vomiting associated with general anesthesia.^[96]^ Moreover, postoperative use of analgesics such as nonsteroidal anti-inflammatory drugs and opioid analgesics can help control nausea and vomiting caused by pain.^[97]^ Pre- and postoperative psychological support, along with the use of antiemetic drugs, can alleviate symptoms of nausea and vomiting, alleviating discomfort.

##### 3.3.3.4. Prevention of nodule rupture

Nodule rupture mostly occurs due to delayed or leaking ablation, typically occurring immediately postoperatively or within hours to days, manifesting as sudden enlargement of the neck or increased pain at the ablation site.^[57]^ Ultrasound or CT imaging may reveal extrathyroidal hemorrhage. Preoperatively, the surgical approach should be determined based on the size of the nodules. For excessively large nodules, ablating them in stages, ensuring thorough ablation in the first instance before subsequent operations, and timely drainage of necrotic material to avoid abscess formation and sinus formation are essential. Intraoperatively, avoiding multiple punctures can minimize inadvertent damage to the thyroid, reducing the risk of injury.^[98]^

##### 3.3.3.5. Reduction of neural damage

A solid grasp of the anatomical structure surrounding the thyroid, precise intraoperative procedures, and minimizing thermal damage are essential for reducing nerve damage. Regular monitoring of the distance between the needle and the nerves surrounding the thyroid is crucial to prevent temporary and permanent nerve damage.^[99]^

### 3.4. Future research directions

#### 3.4.1. Shortcomings in current preventive strategies

While the aforementioned preventive strategies are generally effective in reducing complications, they are not without limitations. For instance, there may be disparities in the training of operators, and there is currently no standardized curriculum or certification system for thyroid RFA operators.

#### 3.4.2. Implications of deficiencies

The lack of standardization may lead to differences in treatment outcomes and the incidence of complications. Furthermore, even with precise techniques and preventive measures, some complications cannot be entirely avoided, necessitating further research in this field.

#### 3.4.3. Direction of future research

Further research and improvement in thyroid RFA need to be approached from multiple perspectives. Firstly, there is a need for further evaluation and monitoring of postoperative symptoms and complications, development of more comprehensive and effective assessment methods, as well as long-term follow-up to monitor the occurrence and progression of postoperative complications.

Secondly, research and development of more advanced and safer surgical techniques and equipment are necessary to reduce damage and risks to the surrounding thyroid tissues during the operation. Concurrently, strengthening operator skills training and technical exchange to enhance surgical quality and safety is crucial.

Additionally, further exploration of effective postoperative care methods and patient education strategies is needed to assist patients in better coping with postoperative recovery issues and considerations, ultimately reducing the incidence of complications.

Finally, through conducting multicenter clinical studies, the safety and effectiveness of thyroid RFA can be further evaluated.^[100]^ Comparisons with other treatment methods can be made, and a large volume of case data can be collected and analyzed to better understand the patterns of postoperative complications and risk factors. This will provide scientific evidence for improving surgical techniques and preventive strategies.

#### 3.4.4. The need for further research

Given the limitations of current preventive strategies, additional research is required to optimize the safety of thyroid RFA. This may involve developing standardized training programs, studying the effectiveness of different preventive measures, and even exploring new technologies to reduce the risk of serious complications.

#### 3.4.5. Potential areas for future research

Potential areas for future research may include utilizing machine learning algorithms to predict complication risks based on patient and nodule characteristics, developing more effective cooling systems to prevent skin burns, and exploring novel materials for RF electrodes to reduce thermal damage to surrounding tissues.

## 4. Conclusion

In conclusion, while thyroid RFA is a safe and effective method for treating benign TN, complications may still occur. Despite the current effectiveness of preventive strategies, there are limitations that require further research to optimize patient outcomes. With advancements in knowledge and technology, it is hoped that the safety of thyroid RFA will continue to improve.

## Author contributions

**Conceptualization:** Junli Qiao.

**Funding acquisition:** Mingqiang Han.

**Investigation:** Shuyan Li.

**Methodology:** Zhanbin Cui.

**Validation:** Lili Zhang.

**Writing – original draft:** Mingqiang Han.

**Writing – review & editing:** Mingqiang Han.
